# High Perceived Stress Predicts Worse Clinical Outcomes in Patients with Stable Coronary Heart Disease

**DOI:** 10.1155/2024/6652769

**Published:** 2024-04-10

**Authors:** Yifan Gao, Yanming Chen, Rong Hu, Cui Tian, Yingyue Zhang, Yanyan Wei, Yajun Shi, Yong Xu, Jing Ma

**Affiliations:** ^1^Department of Cardiology, The First Medical Center of Chinese PLA General Hospital, Beijing 100853, China; ^2^Medical School of Chinese PLA, Beijing 100853, China; ^3^Department of Cardiology and Macrovascular Disease, Beijing Tiantan Hospital, Capital Medical University, Beijing 100070, China; ^4^Senior Department of Cardiology, The Sixth Medical Center, Chinese PLA General Hospital, Beijing 100048, China

## Abstract

**Background:**

High stress is associated with coronary heart disease (CHD), but the impact of perceived stress on prognosis with stable CHD remains unclear. This study investigated the impact of high perceived stress (HPS) on cardiovascular events in stable CHD patients.

**Methods:**

From March 2015 and December 2020, 2215 stable CHD patients were recruited. The Chinese version of the Perceived Stress Scale-14 (CPSS) was used, with follow-up conducted every 6 months until the occurrence of a cardiovascular event or March 31, 2022. Cardiovascular-related events were used as outcomes, including myocardial infarction, unplanned revascularization, stroke, death, or rehospitalization from angina. Patients were divided into HPS (CPSS ≥ 31) and nonhigh perceived stress (NHPS) groups. The Kaplan-Meier survival curves were plotted, and the log-rank test compared the incidence of adverse events after adjusting for sociodemographic, lifestyle, and clinical information.

**Results:**

The recruited CHD population was 59.6 years old on average, 79.6% male, 27.2 points average CPSS, and median follow-up of 47 months. There were 523 HPS patients, with 98 (18.7%) cardiovascular events, and 1692 NHPS patients with 239 (14.1%) cardiovascular events. The log-rank analysis showed that risk of cardiovascular events with HPS was higher than NHPS (*P* = 0.012). After adjusting for demographic, lifestyle, and clinical information, the HPS group had significantly increased risk of events within 24 months (HR 1.369, 95% CI 1.037-1.807, *P* = 0.027), but less impact after 24 months.

**Conclusions:**

HPS predicts subsequent cardiovascular events in patients with stable CHD within 24 months. Therefore, more attention should be given to CHD patients with HPS, which may improve clinical prognosis.

## 1. Introduction

With aging of the global population and acceleration of urbanization, morbidity and mortality due to coronary heart disease (CHD) continue to rise, making CHD an important public health problem worldwide [1]. The occurrence of CHD is not only related to traditional risk factors, such as hypertension, diabetes, hypercholesterolemia, family history, smoking, alcohol consumption, and lack of exercise, but also increasingly to psychosocial stress [2].

The same environmental stressor may be perceived differently by different people, so the magnitude of stress depends largely on an individual's cognitive evaluation of environmental stressors. To objectively and quantitatively measure the degree of stress perception, Cohen et al. developed the original 14-item version of the Perceived Stress Scale (PSS-14) in 1983, which has been widely validated worldwide and translated into different languages [3, 4]. Subsequently, two shortened versions, PSS-10 and PSS-4, were developed, which selected 10 and 4 items from the PSS-14, respectively, that have been used in some studies. The Chinese version of the Perceived Stress Scale-14 (CPSS-14) was developed by Professor Yang Ting-zhong [5] in 2003 with modifications of specific items in the English version of PSS-14 from a Chinese cultural perspective. The validity of CPSS-14 for the general Chinese population has been well established, especially in patients with heart disease [5–7].

High perceived stress (HPS) is a potential risk factor for the occurrence and development of CHD. The INTERHEART study showed that psychosocial stress is associated with increased risk of acute myocardial infarction (AMI) [8]. Moreover, AMI patients with HPS are more likely to experience angina at 1 year and have poorer general health and a higher 2-year mortality rate [9]. Reducing perceived stress levels through stress management training can lower the risk of subsequent clinical events in CHD patients. Blumenthal et al. [10] divided 151 stable CHD patients aged 36 to 84 years into a cardiac rehabilitation+stress management training group, a cardiac rehabilitation group, and a conventional treatment group. Stress management methods included health education, group therapy, and cognitive-behaviour therapy. The study found that compared with the conventional treatment group, the PSS scores of the cardiac rehabilitation+stress management training group and the cardiac rehabilitation group decreased by about 4.2 points and 2.6 points, respectively. During the 5-year follow-up period, both groups had lower rates of cardiovascular events (including death, MI, revascularization, stroke/TIA, and rehospitalization due to worsening angina) than the conventional treatment group. Moreover, the cardiac rehabilitation+stress management training group had lower cardiovascular event rates than the cardiac rehabilitation group alone. Stress management in patients with stable CHD reduces perceived stress levels and cardiovascular clinical events, suggesting that HPS are closely related to cardiovascular events. Our previous cross-sectional study found that among stable CHD patients in China, those with higher education, mental occupation, having children, and/or high-risk drinking habits had higher CPSS scores and, therefore, higher perceived stress levels [11]. However, given the nature of that cross-sectional study, we could not clarify the causal relationship between perceived stress and prognosis of stable CHD. Therefore, the study here was designed to investigate the impact of HPS on cardiovascular events in stable CHD patients.

## 2. Materials and Methods

### 2.1. Study Design

This study was a prospective observational cohort study, which continuously recruited CHD patients who visited our hospital's cardiac rehabilitation clinic from March 2015 to December 2020. After enrolment, all patients were evaluated using CPSS, and subsequent cardiovascular-related events were observed and recorded. This study was approved by the Ethics Committee of the Chinese PLA General Hospital (Ethics Approval No. S2015-125-01), and all participants provided informed consent.

### 2.2. Inclusion and Exclusion Criteria

#### 2.2.1. Inclusion Criteria

1. Patients with a definite diagnosis of stable CHD per the 2013 European Society of Cardiology (ESC) guidelines for the management of stable coronary artery disease [12]
2. Patients who agreed to participate in the CPSS evaluation

#### 2.2.2. Exclusion Criteria

1. Patients with cognitive or language expression disorders
2. Patients who refused to be evaluated by CPSS
3. Patients with acute coronary syndromes

#### 2.2.3. Stress Assessment

The perceived stress of study subjects was evaluated using version 14 of CPSS, which includes 14 items. Each item is scored on a 5-point scale (0 = “never,” 1 = “rarely,” 2 = “sometimes,” 3 = “often,” and 4 = “very often”), with items 4, 5, 6, 7, 9, 10, and 13 being reverse-scored items. The scores of all items are added together to obtain a total score, which ranges from 0 to 56 points: the higher the score, the greater the psychological stress.

#### 2.2.4. Clinical Information Collection

The collection method of baseline data and psychological state evaluations were the same as our previous study [10].

### 2.3. Follow-Up

The investigators followed up subjects by telephone or outpatient clinic visits every 6 months to record whether the patients had cardiovascular events. The date of the first CPSS assessment was considered the starting point for follow-up and survival times, calculated in months. If a subject was lost to follow-up or did not experience a study endpoint event by the time of the final follow-up, their data were considered censored and subjected to truncation processing.

### 2.4. Study Endpoint

The endpoints of this study were cardiovascular events, including MI, unplanned revascularization, stroke, death, or rehospitalization due to worsening angina. The relevant information was obtained from the patient's family members and verified by paper or electronic medical records.

### 2.5. Statistical Analysis

This study was an observational cohort study, and statistical analysis was performed using SPSS 25.0. Statistical charts were drawn using SPSS 25.0 and R software. For quantitative variables, a normality test was performed first, and variables that displayed normal distributions were expressed as mean ± standard deviation (*x* ± SD); variables that did not have normal distributions were expressed as the median and interquartile range (IQR). Qualitative data were expressed as a ratio or percentage. For pairwise comparisons of independent, normally distributed, and homoscedastic quantitative data, a *t*-test (two-tailed unpaired Student's *t*-test) was used, and ANOVA was used for two or more groups. Qualitative data were compared between groups by *χ*^2^ (chi-square test) or Fisher's precision probability test. R software was used to perform survival analysis and to draw survival curves using the Kaplan-Meier method. The difference between groups was first analyzed using the log-rank method for univariate analysis, followed by landmark analysis to adjust for other risk factors or segmented testing. The *P* value was a two-sided test, and *P* < 0.05 was considered statistically significant.

## 3. Results

### 3.1. Baseline Data

**Figure 1:**
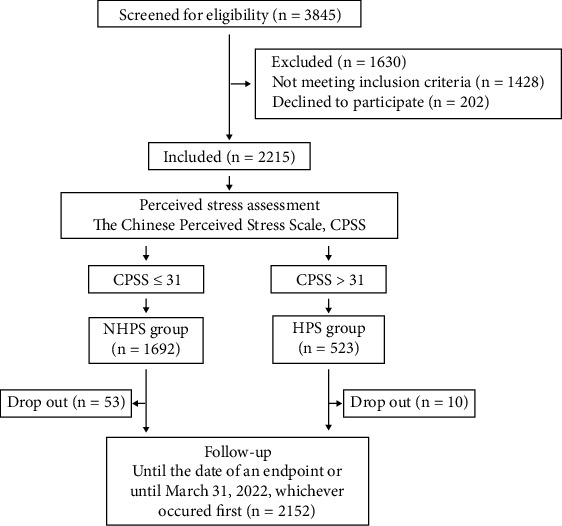
Study flow chart. NHPS: nonhigh perceived stress; HPS: high perceived stress.

A total of 2215 CHD patients were included in the study (Figure 1), with a mean age of 59.6 years and 79.6% male. The sociodemographic characteristics and clinical information of all study subjects can be found in Table S1. The mean CPSS score for all patients was 27.2 ± 6.4 points, with quartile scores of 24, 27, and 31 points. There is no generally accepted cut-off point for the CPSS-14 score. Considering that most mental health-related scales use the cut-off point to redefine the level and degree, this study uses the upper quartile score of stress perception (31 points) as the cut-off point. Then, patients were divided into a HPS group (CPSS score ≥ 31, *n* = 523) and a nonhigh perceived stress (NHPS) group (CPSS score < 31, *n* = 1692). Except for a higher proportion of hypertension in the HPS group, there were no differences in sociodemographic characteristics, lifestyle habits, number of coronary stent implants, complications, anxiety, or depression scores between the HPS and NHPS groups (Table 1).

### 3.2. Endpoint Events

The median follow-up time was 47 months, which ranged from 3 months to 80 months. Sixty-three patients were lost to follow-up (10 in the HPS group and 53 in the NHPS group), with a loss-to-follow-up rate of 2.84%. A total of 337 cases of adverse cardiovascular events were observed during the follow-up period, the most common of which were rehospitalization due to worsening angina (181 cases, 8.2%) and unplanned revascularization (129 cases, 5.8%), followed by acute myocardial infarction (12 cases, 0.5%), death (8 cases, 0.36%), and stroke (7 cases, 0.36%). Compared with the NHPS group, the HPS group had a higher proportion of rehospitalization due to worsening angina, stroke, and total cardiovascular events (Table 2). The Kaplan-Meier analysis was used to plot the cumulative survival curve of cardiovascular events, and overall, the probability of cardiovascular-related clinical events increased with extension of observation time (Figure S1).

### 3.3. Log-Rank Analysis

**Figure 2:**
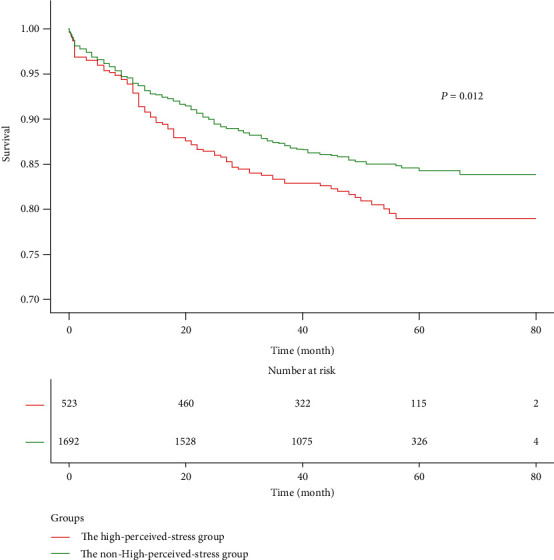
The Kaplan-Meier survival curves of the HPS and NHPS groups. HPS: high perceived stress; NHPS: nonhigh perceived stress.

Log-rank analysis was used to further compare the event-free survival curve and related factors between the two groups. The results showed that the HPS group had a higher risk of events than the NHPS group (*P* = 0.012) (Figure 2).

### 3.4. Landmark Analysis

**Figure 3:**
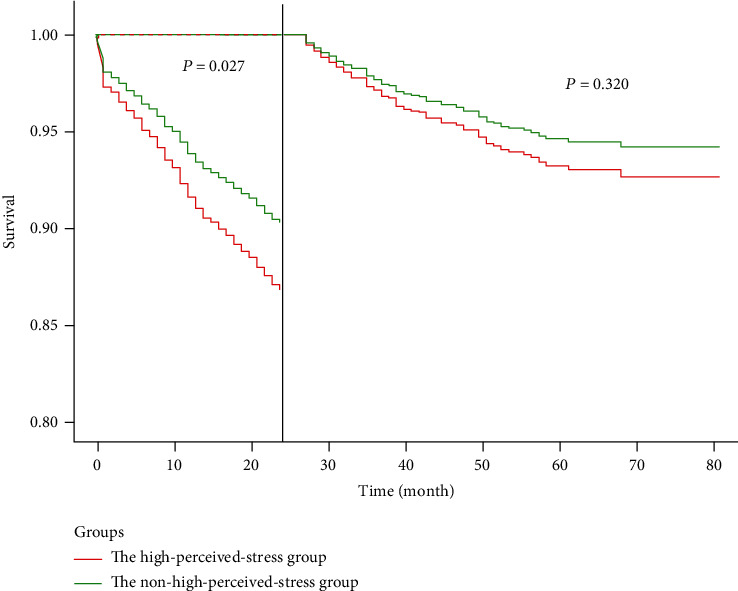
Landmark analysis of the HPS and NHPS groups. HPS: high perceived stress; NHPS: nonhigh perceived stress.

Landmark analysis results showed that after adjusting for sociodemographic characteristics such as age, gender, education level, occupation, smoking, and alcohol consumption, as well as history of myocardial infarction, number of stents implanted, antiplatelet use, hypertension, diabetes, and related factors such as anxiety and depression, HPS still increased the risk of cardiovascular events in patients with CHD (*P* = 0.018). Considering CPSS is time-sensitive, we measured the incidence of adverse events within 24 months and 24 months to the end of follow-up, respectively. The study found that within the 0–24-month time window, CHD patients with HPS had a higher risk of cardiovascular events compared with the NHPS group (HR 1.369, 95% CI 1.037-1.807, *P* = 0.027); however, after 24 months, the impact of perceived stress on patient prognosis decreased, and there was no statistical difference between the two groups (Table S2 and Figure 3).

### 3.5. Subgroup Analysis

To investigate whether the predictive value of perceived stress on clinical outcomes differs among CHD patients with different characteristics, we conducted a subgroup analysis on relevant populations (Figure S2). The results showed that there was no interaction between age, gender, history of myocardial infarction, hypertension, or diabetes with perceived stress, and the impact of perceived stress on prognosis did not differ among different subgroups.

## 4. Discussion

Psychosocial states, like perceived stress, are known to play a role in health, though direct physiological mechanisms are not fully elucidated. In this study, we investigated the relationship between stress perception and CHD prognosis, which may provide new warning indicators for the management of CHD. This real-world research studied 2215 stable CHD patients with a median follow-up time of 47 months and a maximum of 80 months. It is not only the largest study to date in evaluating the impact of perceived stress on the prognosis of stable CHD but also the longest prospective study with follow-up. The study found that the average CPSS score for stable CHD patients was 27.2 points, and stable CHD patients with HPS (CPSS ≥ 31 points) were at higher risk of subsequent cardiovascular events. The risk persisted even after adjusting for traditional cardiovascular risk factors, and this risk persisted and remained significant for 24 months.

The level of perceived stress is closely related to the risk of cardiovascular events in CHD patients, which is consistent with the results of previous clinical studies conducted by Yin et al. [13]. That study included 241 CHD patients in China, with an average follow-up time of 26 months, and found that PSS scores (using PSS-10) were related to major adverse cardiovascular events. However, only 77 of those subjects were diagnosed with stable CHD, and the remaining 164 were acute coronary syndrome patients. The effect of acute stress on perceived stress scores cannot be ruled out [14]. In contrast, our study focused only on stable CHD patients with a smaller sample error, a larger sample size, the full version of CPSS, and a longer follow-up period. The results showed that a high level of perceived stress was an independent risk factor for adverse cardiovascular events in CHD patients.

CPSS does have significant temporal limitations in predicting adverse events in CHD prognosis. It is noteworthy that our results are not entirely consistent with the results of Pimple et al.'s study [15]. That study included 662 stable CHD patients, 28% of whom were female and 30% were black. The average follow-up time was 2.8 years, and there was no association between PSS scores and CVD events, though a higher PSS score was associated with higher rates of cardiovascular events in female CHD patients but not in male. The difference with those study results may be related to different races being studied, which would require further research.

Considering the timeliness of perceived stress scores, we separately tested the predictive value of CPSS for short-term and long-term prognosis. We found that CPSS within 0-24 months had a greater predictive significance for adverse events in stable CHD, while the impact of perceived stress on patient prognosis diminished after 24 months, with no statistical difference between the two groups. This is because the level of stress perception in CHD patients is not fixed and can change with time, environment, and cognition of disease [16, 17]. One study found that the level of perceived stress decreased over time in 2358 young and middle-aged patients with acute MI [14]. This suggests that a CHD patient's level of perceived stress should be reviewed in a timely manner to understand any changes pertinent to that patient's prognosis. Although stress levels change, our study shows that a simple screening of stress levels can help predict adverse events in CHD within 24 months.

Further analysis of the clinical characteristics reveals that compared with NHPS patients, the proportion of patients with hypertension was higher in the HPS group (61.2% vs. 54.7%, *P* = 0.009), which is consistent with previous studies showing that HPS was significantly associated with a 15% increase in hypertension development [18, 19]. The incidence of stroke in HPS was significantly higher than that in the NHPS group, which may be related to the higher proportion of hypertension in the HPS group [20, 21].

HPS has been associated with adverse cardiovascular events through multiple mechanisms, including coronary vasoconstriction and reduced coronary blood flow, platelet reactivity, endogenous coagulation system activation, endothelial dysfunction, inflammation, and metabolic risk factors [22, 23]. Stress may also be associated with unhealthy lifestyle habits such as smoking, drinking, and sedentary behaviour. In addition to behavioural factors, persistent stress may increase the risk of metabolic syndrome, which is further associated with increased incidence of CHD risk factors such as obesity, hypertension, high cholesterol and triglyceride levels, and type 2 diabetes [24]. However, stress is a modifiable risk factor.

Reducing perceived stress levels through stress management training can lower the risk of subsequent clinical events in CHD patients [10]. Other studies have also shown that improving stress-coping skills can improve the quality of life in CHD patients [25]. Cognitive behaviour training and meditation training for CHD patients can reduce the risk of recurrent cardiovascular events and prolong life expectancy after acute MI [26, 27]. In summary, stress management could reduce CPSS scores and improve the prognosis of CHD patients. This suggests that stress management training should be routinely included in cardiac rehabilitation programs of CHD patients.

It should be emphasized that the CPSS is not a diagnostic tool and is generally used only to measure the perceived stress level of an evaluated individual. The scale itself does not have a fixed threshold, and the purpose of this study is not to explore a clear threshold, so patients were simply grouped based on the median and upper quartile of this study to identify the clinical prognosis differences between stable CHD patients with HPS levels and NHPS levels in China. Using landmark analysis to correct for other influencing factors and segmented analysis of the risk of clinical events occurring over 2 years or more, high CPSS scores were found to increase the risk of adverse clinical events in stable CHD patients within 24 months. This suggests that when managing CHD patients, the level of perceived stress should be considered, and a simple screening tool should be used for dynamic assessment to provide personalized stress management training for patients with high levels of perceived stress to improve prognosis.

### 4.1. Limitations

This study has several limitations. Firstly, it is a single-center study, and it is inevitable that there is a certain selection bias. We are continuing this observation in more centers to improve the representativeness of the sample. Secondly, as CPSS does not have a clear cut-off value, we used the upper quartile of the CPSS score of the study population to define HPS, which leads to some uncertainty in extending the study results to other populations. Finally, the enrolled population consisted mainly of middle-aged and elderly stable CHD patients in China, and the generalizability to other populations in other parts of the world needs further study.

## 5. Conclusion

HPS can predict worse clinical outcomes in patients with stable CHD, especially within 24 months. Therefore, more attention should be given to CHD patients with HPS, which may improve their clinical prognosis with appropriate interventions.

## Figures and Tables

**Table 1:** Social demography factors of HPS and NHPS groups.

Variable, *N* (%)	NHPS group (*n* = 1692)	HPS group (*n* = 523)	*P*
Age, mean (SD) (y)	59.4 ± 10.0	60.1 ± 10.3	0.594
Male	1357 (80.2)	407 (77.8)	0.237
Education level			0.357
Senior high school and below	839 (49.6)	268 (51.2)	
College	749 (44.3)	229 (43.8)	
Above college	104 (6.1)	26 (5.0)	
Having children	1655 (97.8)	515 (98.5)	0.352
Married	1677 (99.1)	517 (98.9)	0.599
Mental occupation	1173 (69.3)	357 (68.3)	0.645
Regular exercise	1259 (74.4)	371 (70.9)	0.115
Risky alcohol drinking	1469 (86.8)	448 (85.7)	0.497
Current smoker	250 (14.8)	84 (16.1)	0.236
Sleep disorder	1269 (75.0)	388 (74.2)	0.708
History of MI	527 (31.1)	159 (30.4)	0.747
Number of stents	1.4 ± 1.3	1.4 ± 1.2	0.467
Antiplatelet therapy			0.181
No	403 (23.8)	123 (23.5)	
SAPT	488 (28.8)	172 (32.9)	
DAPT	801 (47.3)	228 (43.6)	
Hypertension	925 (54.7)	320 (61.2)	0.009⁣^∗^
Hyperlipidemia	793 (46.9)	256 (48.9)	0.405
Diabetes	459 (27.1)	139 (26.6)	0.804
Cerebrovascular disease	25 (1.5)	14 (2.7)	0.068
PHQ-9	5.2 ± 4.0	5.2 ± 3.6	0.822
GAD-7	4.2 ± 4.3	4.1 ± 4.3	0.389

NHPS: nonhigh perceived stress; HPS: high perceived stress; MI: myocardial infarction; SAPT: single antiplatelet therapy; DAPT: dual antiplatelet therapy; PHQ-9: 9-item patient health questionnaire; GAD-7: 7-item generalized anxiety disorder scale. ⁣^∗^*P* < 0.05.

**Table 2:** Cardiovascular events during follow-up.

Endpoints, *N* (%)	NHPS group (*n* = 1692)	HPS group (*n* = 523)	Total	*P* value
Rehospitalization^†^	126 (7.4%)	55 (10.5%)	181	0.025⁣^∗^
Unplanned revascularization	93 (5.5%)	36 (6.8%)	129	0.237
Myocardial infarction	11 (0.6%)	1 (0.2%)	12	0.315
Death	7 (0.4%)	1 (0.2%)	8	0.689
Stroke	2 (0.1%)	5 (0.9%)	7	0.010⁣^∗^
Total	239 (14.1%)	98 (18.7%)	337	0.009⁣^∗^

^†^Rehospitalization refers to rehospitalization due to worsening angina. HPS: high perceived stress; NHPS: nonhigh perceived stress. ⁣^∗^*P* < 0.05.

## Data Availability

The data are available from the corresponding authors on reasonable request.

## Supplementary Materials

Supplementary Materials Table S1: social demography factors of the subjects. Figure S1: cumulative survival curve. Table S2: multivariate landmark analysis. Figure S2: subgroup analysis.
