# Supplementary material for: High Perceived Stress Predicts Worse Clinical Outcomes in Patients with Stable Coronary Heart Disease
Source: Depress Anxiety. 2024 Apr 10;2024:6652769. doi: 10.1155/2024/6652769 (PMC11919031; doi:10.1155/2024/6652769)
Supplement: Supplementary Materials — Table S1: social demography factors of the subjects. Figure S1: cumulative survival curve. Table S2: multivariate landmark analysis. Figure S2: subgroup analysis. [file 6652769.f1.docx]

## Supplementary Materials

Table S1 Social demography factors of the subjects

| **Social Demography Factor** | | **N** | **%** |
| --- | --- | --- | --- |
| **Age** | <45 | 160 | 7.2 |
|  | 45-64 | 1317 | 59.5 |
|  | 65-84 | 727 | 32.8 |
|  | ≥85 | 11 | 0.4 |
| **Gender** | Male | 1764 | 79.6 |
|  | Female | 451 | 20.4 |
| **Region** | North | 1388 | 62.7 |
|  | South | 827 | 37.3 |
| **Education level** | Senior high school and below | 1107 | 50.0 |
|  | College | 978 | 44.2 |
|  | Above college | 130 | 5.9 |
| **Having children** | Yes | 2170 | 98.0 |
|  | No | 45 | 2.0 |
| **Marital status** | Married | 2194 | 99.0 |
|  | Other (single, divorced, or widowed) | 21 | 1.0 |
| **Occupation** | Mental | 1530 | 69.1 |
|  | Physical | 685 | 30.9 |
| **Regular exercise** | Yes | 1630 | 73.6 |
|  | No | 585 | 26.4 |
| **Risky alcohol drinking** | Yes | 298 | 13.5 |
|  | No | 1917 | 86.5 |
| **Current Smoker** | Yes | 334 | 15.1 |
|  | No | 1881 | 84.9 |
| **Comorbidity** |  |  |  |
| **Number of stents** | 0 | 508 | 22.9 |
|  | 1 | 901 | 40.7 |
|  | 2 | 462 | 20.9 |
|  | ≥3 | 344 | 15.5 |
| **Hypertension** | Yes | 1245 | 56.2 |
|  | No | 970 | 43.8 |
| **Hyperlipemia** | Yes | 1049 | 47.4 |
|  | No | 1166 | 52.6 |
| **Diabetes** | Yes | 598 | 27.0 |
|  | No | 1617 | 73.0 |
| **Cerebrovascular disease** | Yes | 39 | 1.8 |
|  | No | 2176 | 98.2 |
| **Other diseases** | Yes | 119 | 7.1 |
|  | No | 2096 | 92.9 |
| **Sleep disorder** | Yes | 1657 | 74.8 |
|  | No | 558 | 25.2 |
| **Depressive state** | Yes | 817 | 36.9 |
|  | No | 1398 | 63.1 |
| **Anxiety state** | Yes | 1113 | 50.2 |
|  | No | 1102 | 49.8 |

##
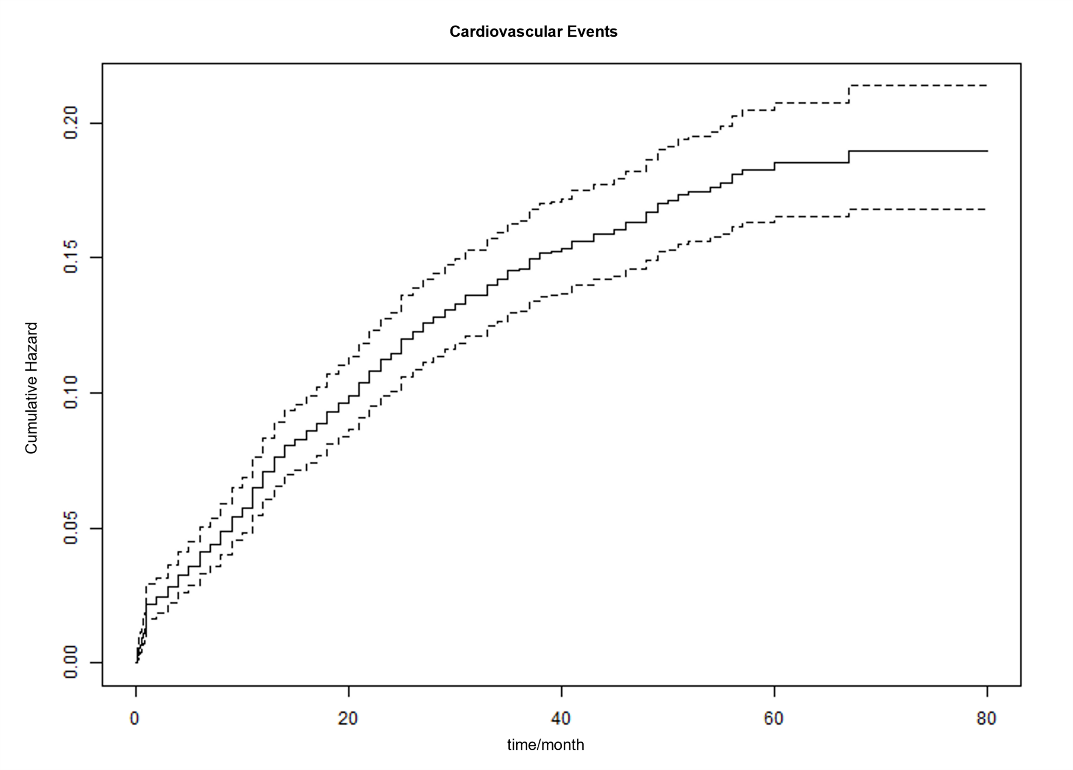


Figure S1 Cumulative survival curve

Table S2 Multivariate landmark analysis*

|  | **Coef** | **Se Coef** | **Z** | **HR (95%CI)** | ***P*** |
| --- | --- | --- | --- | --- | --- |
| **0-80 m** |  |  |  |  |  |
| HPS | 0.283 | 0.120 | 2.359 | 1.328 (1.049-1.680) | 0.018 |
| Age | -0.001 | 0.007 | -0.181 | 0.999 (0.985-1.013) | 0.856 |
| Occupation | -0.118 | 0.150 | -0.792 | 0.888 (0.663-1.191) | 0.428 |
| Female | 0.400 | 0.122 | 3.278 | 1.492 (1.174-1.894) | 0.001 |
| History of MI | 0.163 | 0.116 | 1.400 | 1.177 (0.937-1.478) | 0.161 |
| **0-24 m** |  |  |  |  |  |
| HPS | 0.314 | 0.142 | 2.215 | 1.369 (1.037-1.807) | 0.027* |
| Age | 0.003 | 0.007 | 0.463 | 1.003 (0.990-1.016) | 0.643 |
| Occupation | -0.131 | 0.1471 | -0.889 | 0.877 (0.658-1.171) | 0.374 |
| Female | 0.305 | 0.149 | 2.043 | 1.357 (1.013-1.819) | 0.041* |
| History of MI | 0.351 | 0.134 | 2.624 | 1.421 (1.093-1.847) | 0.009* |
| **24-80 m** |  |  |  |  |  |
| HPS | 0.227 | 0.228 | 0.996 | 1.255 (0.803-1.962) | 0.320 |
| Age | -0.001 | 0.011 | -0.054 | 0.999 (0.979-1.021) | 0.957 |
| Occupation | -0.278 | 0.225 | -1.238 | 0.757 (0.487-1.176) | 0.216 |
| Female | 0.542 | 0.218 | 2.491 | 1.720 (1.123-2.634) | 0.013* |
| History of MI | -0.357 | 0.237 | -1.505 | 0.700 (0.439-1.114) | 0.132 |

*Adjusted for age, gender, education level, occupation, smoking status, alcohol consumption, history of myocardial infarction, number of implanted stents, antiplatelet therapy, heart failure, hypertension, diabetes, and anxiety and depression; HPS: high-perceived-stress; NHPS: non-high-perceived-stress; MI: myocardial infarction; **P*<0.05


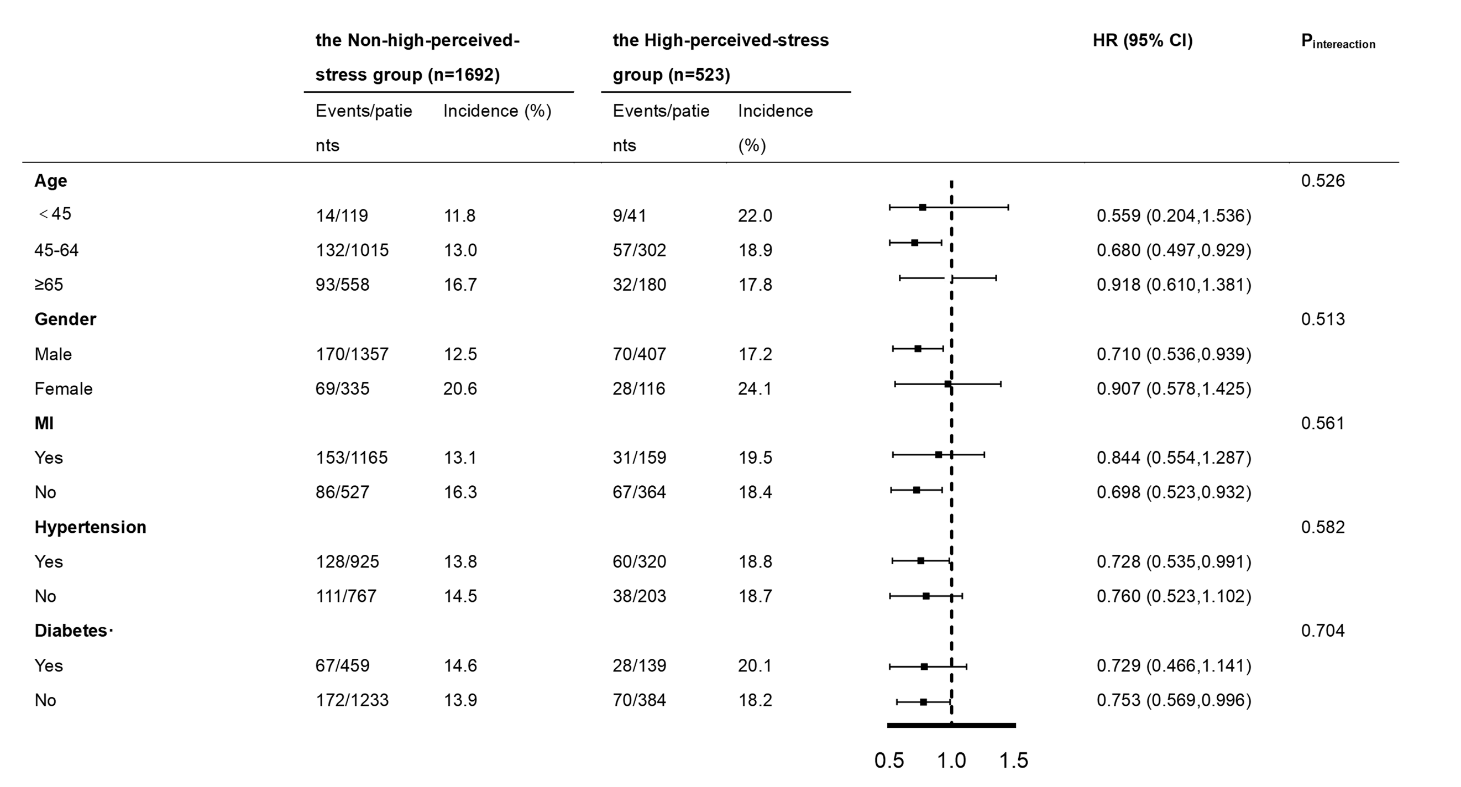


Figure S2 Subgroup analysis
